# Role of plasma EBV-DNA load and EBER status on newly diagnosed peripheral T-cell lymphoma

**DOI:** 10.1007/s00432-024-05702-9

**Published:** 2024-04-08

**Authors:** Jing Chen, Jie Zhou, Fei Cheng, Donghe Chen, Fangshu Guan, Enfan Zhang, Jingsong He, Zhen Cai, Yi Zhao

**Affiliations:** 1 Bone Transplantation Center, The First Affiliated Hospital, Zhejiang University School of Medicine, Hangzhou, Zhejiang Province China; 2 Pathology Department, The First Affiliated Hospital, Zhejiang University School of Medicine, Hangzhou, Zhejiang Province China; 3 Department of Nuclear Medicine, The First Affiliated Hospital, Zhejiang University School of Medicine, Hangzhou, Zhejiang Province China

**Keywords:** Epstein-Barr virus, Prognosis, Treatment, Peripheral T-cell lymphoma

## Abstract

**Purpose:**

To explore the prognostic and therapeutic role of Epstein-Barr Virus (EBV) on peripheral T-cell lymphoma (PTCL).

**Methods:**

Totally 262 newly diagnosed PTCL patients who were hospitalized from January 2014 to December 2022 were retrospectively enrolled. Molecular analysis included 31 eligible patients. EBV-encoded RNA (EBER) presence in tumor tissue and EBV DNA levels in patients at baseline (DNA1) and after 4 cycles of chemotherapy (DNA4) were assessed.

**Results:**

Our findings revealed that the EBER-positive cohort exhibited significant differences compared to counterparts in overall survival (OS, *P* = 0.047) and progression-free survival (PFS, *P* = 0.009). Both DNA1 and DNA4 were significantly associated with inferior OS. Multivariate analysis demonstrated that DNA4 independently affected PTCL prognosis for OS (hazard ratio = 5.1617; 95% confidence interval 1.1017–24.1831; *P* = 0.037). Treatment with the cyclophosphamide, doxorubicin, vincristine, and prednisone (CHOP) plus azacytidine regimen showed a better OS compared to CHOP or CHOP plus etoposide for patients with partially positive EBER and EBER positive statuses (*P* = 0.192), although the improvement was not statistically significant. This study delineated the genetic paradigm of PTCL, comparing genetic differences by EBV status and found that EBER partially positive plus positive patients were more likely to have DNMT3A (*P* = 0.002), RHOA^G17V^ (*P* = 0.023), and TET2 mutations (*P* = 0.032).

**Conclusion:**

EBER, DNA1, and DNA4 emerged as sensitive markers for prognosis. CHOP plus azacytidine might present a preferable option for PTCL patients with DNA methylation due to EBV infection.

**Supplementary Information:**

The online version contains supplementary material available at 10.1007/s00432-024-05702-9.

## Introduction

Peripheral T-cell lymphomas (PTCLs), a group of heterogeneous T-cell neoplasms, originate from post-thymic lymphocytes and constitute approximately 15% of non-Hodgkin lymphomas, including peripheral T-cell lymphoma, not otherwise specified (PTCL-NOS), angioimmunoblastic T-cell lymphoma (AITL), nodal T-follicular helper (TFH) cell lymphoma (PTCL-TFH), systemic anaplastic large cell lymphoma (sALCL), among others (Alaggio et al. 2022). ALCLs, classified based on the anaplastic lymphoma kinase (ALK) status, comprise two groups. Apart from ALK + ALCLs, most PTCLs exhibit a grim prognosis, with a 5-year overall survival (OS) ranging from 10 to 51% (Zhang et al. 2016; Rodríguez et al. 2021; Mina and Pro 2022).

Recognizing patients at high risk and selecting appropriate therapeutic regimens are essential. Epstein–Barr virus (EBV) is a ubiquitous DNA virus belonging to the gamma subfamily of herpesviruses, associated with several lymphoma subtypes, such as Burkitt's lymphoma (BL) and extranodal NK/T-cell lymphoma (ENKTL) (Bednarska et al. 2024; Damania et al. 2022; Lopez et al. 2022; Xiong et al. 2020). EBV-associated lymphomas are characterized by the presence of EBV-encoded RNA (EBER) in tumor tissue. However, the effect of EBV on PTCL remains controversial. Some studies (Dupuis et al. 2006; Weisenburger et al. 2011; Kim et al. 2021) have demonstrated poor survival outcomes in patients with EBV infection, while others have presented contrary views (Haverkos et al. 2017; Shen et al. 2022). Additionally, only a few studies have compared different EBV detection methods, such as EBER using in situ hybridization (ISH) in neoplasms and EBV DNA polymerase gene in peripheral blood using polymerase chain reaction (PCR) (Kim et al. 2021; Shen et al. 2022; Zeng et al. 2022).

The optimal first-line chemotherapy regimen for PTCL remains undefined. The addition of etoposide to cyclophosphamide, doxorubicin, vincristine, and prednisone (CHOP/E) has been proposed to enhance clinical benefit, but its therapeutic efficacy is disputed (Deng et al. 2019; Liu et al. 2018; Jia et al. 2016). Some studies have indicated that EBV infection typically leads to extensive methylation, contributing to immune escape and disease progression in conditions such as gastric cancers, nasopharyngeal carcinoma, and breast cancer (Matsusaka 2014; Usui et al. 2021; Peng et al. 2016; Abdallah et al. 2018). Hypomethylating agents, such as azacytidine (AZA), have the potential to reduce genomic methylation levels and reactivate hypermethylated tumor suppressor genes. Therefore, targeting DNA methylation may represent a novel therapeutic strategy for EBV-associated diseases (Zhang et al. 2022).

This study aimed to investigate the frequency, clinical features, and survival outcomes of PTCL with EBV, compare the use of two methods for detecting EBV (EBER vs. EBV DNA), and monitor dynamic changes in EBV DNA. This study also found evidence suggesting that CHOP plus AZA may offer promising treatment efficacy for EBV-associated patients, along with relative genetic analysis.

## Methods

### Patients

We retrospectively collected data from 262 eligible and newly diagnosed PTCL patients between January 2014 and December 2022 at our hospital for survival analysis. During this period, 31 patients were enrolled for molecular analysis. Detailed inclusion and exclusion criteria are provided in the supplementary material.

Clinical characteristics, plasma EBV DNA titers before chemotherapy (DNA1) and after 4 cycles of chemotherapy (DNA4), response status, and survival outcomes were recorded. Lymphoma response criteria included overall response rate (ORR): complete remission (CR) plus partial remission (PR), stable disease (SD), and progressive disease (PD). Recurrence was defined as PD after more than 6 months of CR. The follow-up deadline was June 30, 2023.

This study protocol received approval from the Research Ethics Committee, and informed consent was obtained from all patients or their immediate relatives.

### EBV DNA and EBER detection

The presence of EBV-specific small RNAs was assessed using ISH. EBER oligonucleotides were applied to formalin-fixed paraffin-embedded sections following the Inform EBER Probe Assay Protocol. EBER positivity was defined as the presence of EBER-positive neoplastic cells in the highest density region exceeding 50/high-power field, as per relevant literature (Shen et al. 2022; Zeng et al. 2022). EBER partial positivity (classified as EBV negative) indicated the presence of EBER-positive neoplastic cells but not exceeding 50% in high-power field.

Plasma samples collected before treatment were utilized for EBV DNA analysis. DNA was extracted from plasma samples using the EBV PCR Fluorescence Quantitative Diagnostic Kit. Copy numbers were analyzed using a standard curve, with 500 copies/mL defined as the critical value based on previous studies (Kim et al. 2021; Shen et al. 2022; Zeng et al. 2022; Qiu et al. 2022). If EBV DNA was less than 500 copies/mL, it was recorded as 0. Given the significance of interim evaluation [such as interim positron emission tomography/computed tomography (Wang et al. 2017)] for prognosis, DNA levels were also collected after 4 courses of treatment. The optimal cut-off points for DNA1 and DNA4 (> 6750 copies/mL) were determined using receiver operating curve (ROC) analysis.

### Comparison of three treatment options

To explore the optimal regimen for PTCL with EBV infection, survival outcomes for patients receiving different first-line treatments were compared. Considering the typical characteristic of EBV infection (extensive methylation), patients were classified into three groups: Option A (CHOP plus AZA cohort), Option B (CHOPE cohort), and Option C [CHOP cohort including cyclophosphamide, vincristine, prednisolone (COP), and cyclophosphamide, epirubicin, vindesine, and prednisolone (CDOP)].

### Next generation sequencing (NGS)

NGS covered the exons, fusion-related intron regions, and alternative splicing regions of 103 genes related to T/NK cell lymphoma, based on authoritative TCGA database, NCCN guidelines, and 2016 WHO consensus. The detection platform used was illumina Hiseq/MiSeqDx/NextSeq. Relevant gene expression profiling was obtained from the medical record system.

### Statistical analysis

Continuous variables were transformed into categorical variables using ROC analysis or reports from relevant literature. Chi-squared or Fisher’s exact tests were used for categorical variables, while the Mann–Whitney *U* test and Student’s t test were used for continuous variables. Competing risk analyses were performed according to the Fine–Gray method, assessing relapse and death as competing events. Agreement between EBER status and DNA level was characterized using the kappa statistic and measures of sensitivity, specificity, positive predictive value (PPV), and negative predictive value (NPV). Endpoint events (OS; progression-free survival, PFS) were analyzed using Kaplan–Meier methods, with a log-rank test used for comparison. Potential factors were initially analyzed by univariate analysis. Factors with *P* < 0.1 in univariate analysis or deemed important clinical factors according to the Cox proportional hazard regression model were included in multivariate analysis. Reported p values were two-sided, with *P* < 0.05 considered statistically significant.

## Result

### Baseline characteristics

A total of 262 patients with PTCL were enrolled in this retrospective study, and the baseline characteristics according to different EBER statuses are presented in Table 1. Of these patients, 147 had AITL, 56 had PTCL-NOS, 19 had PTCL-TFH, and 40 had sALCL. The median age was 60.1 years (range 15–87), with 21.4% of patients having Eastern Cooperative Oncology Group performance status scores (ECOG PS) ≥ 2. Two hundred and thirty-one patients had advanced disease stage (stage III/IV), including 52 with bone marrow infiltration (BMI). Approximately 7% (19/262) of cases among all patients had extranodal sites (ENSs) ≥ 2. Furthermore, patients with AITL (14.3%, 21/147) and PTCL-TFH (15.8%, 3/19) were more prone to EBV infection compared to those with PTCL-NOS (8.9%, 5/56) and sALCL (2.5%, 1/40).

**Table 1:** Baseline characteristics in PTCL patients by the status of EBV

Characteristic	Total	EBER negative (*n* = 232)	EBER positive (*n* = 30)	*P*-value
Female, no. (%)	109 (41.6)	98 (42.2)	11 (36.7)	0.560
Age > 60 years, no. (%)	148 (56.5)	127 (54.7)	21 (70.0)	0.113
Stage III/IV, no. (%)	231 (88.2)	205 (88.4)	26 (86.7)	0.432
ENSs ≥ 2, no. (%)	19 (7.3)	19 (8.2)	0 (0)	0.142
B symptom, no. (%)	118 (45.0)	103 (44.4)	15 (50.0)	0.566
BMI, no. (%)	52 (19.8)	46 (19.8)	6 (20.0)	1.000
ECOG PS ≥ 2, no. (%)	56 (21.4)	46 (19.8)	10 (33.3)	0.090
Elevated LDH, no. (%)	144 (59.0)	128 (59.5)	16 (55.2)	0.647
IPI, no. (%)	n = 244	n = 215	n = 29	0.476
0–1 (a Low IPI)	45 (18.4)	39 (18.2)	6 (20.6)	
2 (a Low-Intermediate IPI)	96 (39.3)	88 (40.9)	8 (27.6)	
3 (a High-Intermediate IPI)	76 (31.1)	66 (30.7)	10 (34.5)	
4–5 (a High IPI)	27 (11.1)	22 (10.2)	5 (17.2)	
PIT, no. (%)	*n* = 244	*n* = 215	*n* = 29	0.386
0	32 (14.8)	32 (14.9)	4 (13.8)	
1	79 (29.4)	71 (33.0)	8 (27.6)	
2	89 (36.5)	80 (37.2)	9 (31.0)	
3–4	40 (16.4)	32 (14.9)	8 (27.6)	
Subtypes, no. (%)	*n* = 262	*n* = 232	*n* = 30	0.131
PTCL-NOS	56 (21.4)	51 (22.0)	5 (16.7)	
AITL	147 (56.1)	126 (54.3)	21 (70.0)	
PTCL-TFH	19 (7.3)	16 (6.9)	3 (10.0)	
ALCL	40 (15.3)	39 (16.8)	1 (3.3)	
First-line treatment, no. (%)	n = 262	n = 232	n = 30	0.392
CHOP and CHOP-like	125 (47.7)	110 (47.4)	15 (50.0)	
CHOPE	63 (24.0)	58 (25.0)	5 (16.7)	
CHOP + AZA	22 (8.4)	19 (8.2)	3 (10.0)	
Combined with chidamide	9 (3.4)	9 (3.9)	0 (0.0)	
Combined with brentuximab vedotin	15 (5.7)	14 (6.0)	1 (3.3)	
Combined with lenalidomide	10 (3.8)	7 (3.0)	3 (10.0)	
Others	18 (6.9)	15 (6.5)	3 (10.0)	

*PTCL* peripheral T-cell lymphoma, *EBV* Epstein-Barr virus, *EBER* EBV-encoded RNA, *ENSs* extranodal sites, *BMI* bone marrow infiltration, *ECOG PS* Eastern Cooperative Oncology Group performance status, *LDH* lactate dehydrogenase, *IPI* International Prognostic Index, *PIT* prognostic index for PTCL, *PTCL-NOS* peripheral T-cell lymphoma not otherwise specified, *AITL* angioimmunoblastic T-cell lymphoma, *PTCL-TFH* nodal T-follicular helper (TFH) cell lymphoma, *ALCL* anaplastic large cell lymphoma, *CHOP* cyclophosphamide doxorubicin vincristine and prednisone, *CHOPE* etoposide plus CHOP, *AZA* azacytidine

No significant differences were observed in sex, stage, symptoms, BMI, elevated LDH, IPI, or PIT between the EBER-positive and EBER-negative cohorts. There were moderate but non-statistically significant associations between the EBV cohorts and ECOG PS (*P* = 0.090). EBER-positive patients tended to be older (average age, *P* = 0.012) and have lower levels of albumin (*P* = 0.022) than their counterparts.

The majority of eligible patients received CHOP/CHOP-like therapy as first-line treatment. In addition, 23 patients underwent autologous hematopoietic cell transplantation (auto-HCT), and 1 patient received allogeneic hematopoietic stem cell transplantation (allo-SCT). Only 2 patients received radiotherapy during treatment.

### Association between EBV DNA and EBER status

**Fig. 1:**
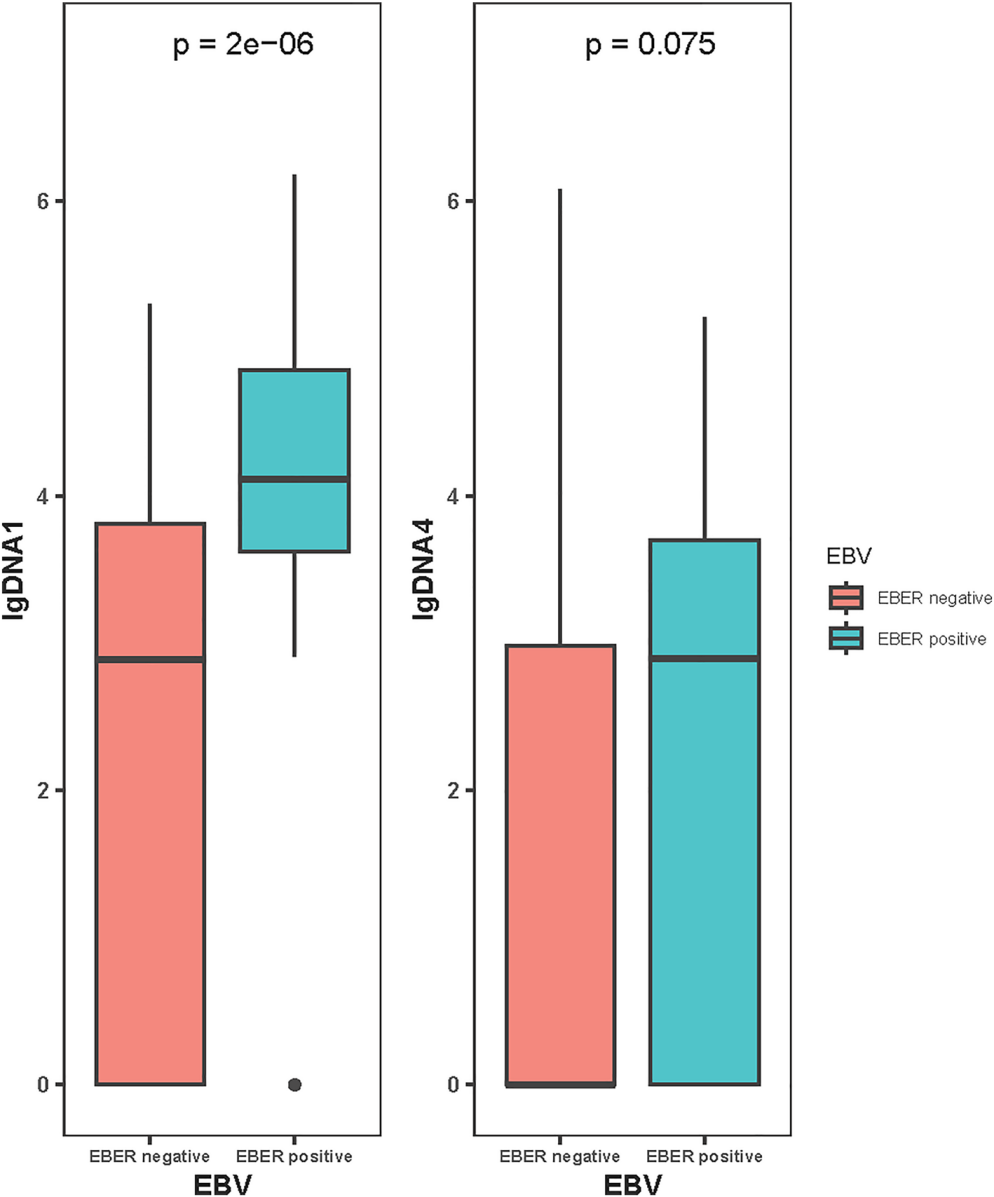
EBV DNA quantification analysis before and after treatment. When DNA is 0, lgDNA takes the value 0; other DNA levels are greater than 1 (DNA here is a continuous variable). *EBV* Epstein–Barr virus, *EBER* EBV-encoded RNA, *DNA1* the EBV DNA level of patient at baseline, *DNA4* the EBV DNA level of patient after 4 cycles of chemotherapy

To investigate the relationship between EBV DNA and EBER status, diagnostic tests were conducted. A total of 167 cases were included, with 19 patients confirmed with EBV infection by EBER and 50 confirmed by DNA1 (> 6750 copies/mL). The kappa statistic was 0.254, indicating poor consistency. PPV is 26% and NPV is 94.9%. The sensitivity and specificity of DNA1 as a surrogate for EBER were 68.4% and 75.0%, respectively (see Table S1). These results demonstrate poor consistency between DNA1 and EBER status. Out of 124 patients tested by PCR after 4 cycles of treatment, those with EBER positivity were more likely to have a high level of DNA1 (*P* = 0.000) at diagnosis. However, this tendency was not significant after 4 cycles of treatment (*P* = 0.075) (see Fig. 1).

### Response evaluations

To investigate the possibility that patients with EBER negativity achieve remission more easily, response evaluations were conducted (see Table S2). In the EBER-negative cohort, the ORR was 51.2%, with 33 cases achieving SD and 80 cases experiencing PD. In the EBER-positive cohort, the ORR was 30.0%, with 10 cases achieving SD and 11 cases experiencing PD. A significant difference (*P* = 0.015) in ORR was observed between EBER-positive and EBER-negative patients. Although not statistically significant, patients with lower levels of DNA1 (≤ 6750 copies/mL) tended to achieve ORR more easily (*P* = 0.078).

The cumulative incidence function of relapse was comparable between the two groups based on EBER status when death was considered as a competing event. EBER status failed to predict relapse in competing risk models (*P* = 0.496).

### Survival analysis

**Fig. 2:**
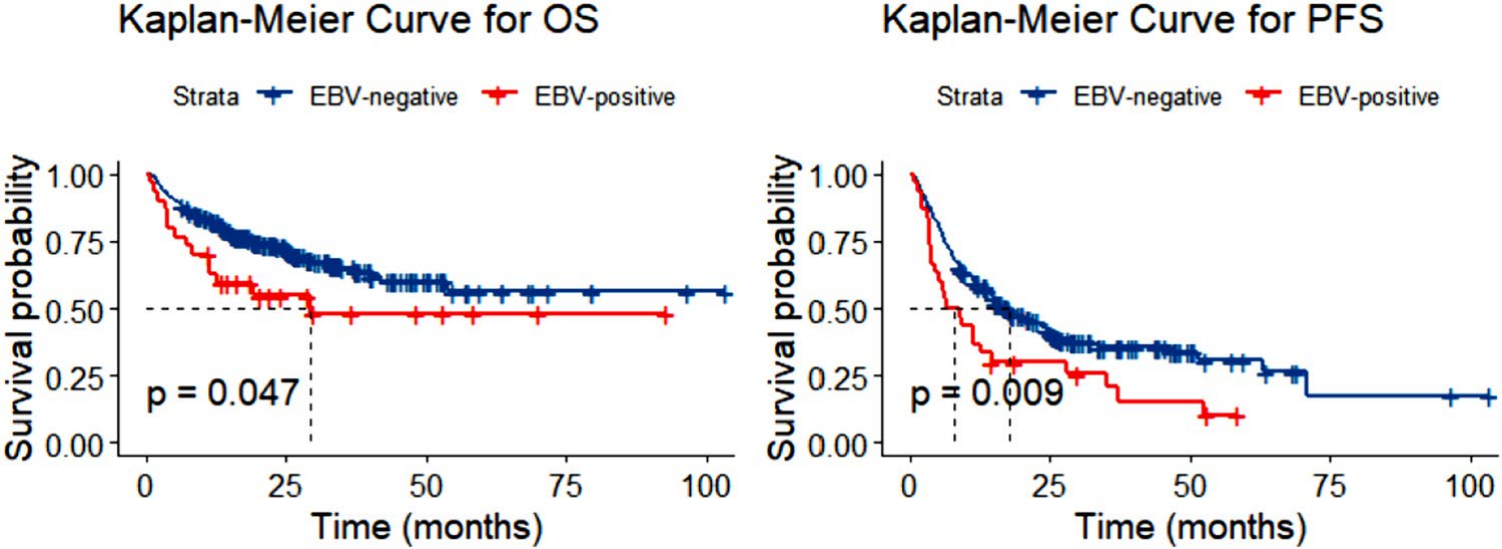
Overall survival (OS) (**A**) and progression-free survival (PFS) (**B**) in patients with PTCL according to status of EBER. *PTCL* peripheral T-cell lymphoma, *EBV* Epstein–Barr virus, *EBER* EBV-encoded RNA

**Fig. 3:**
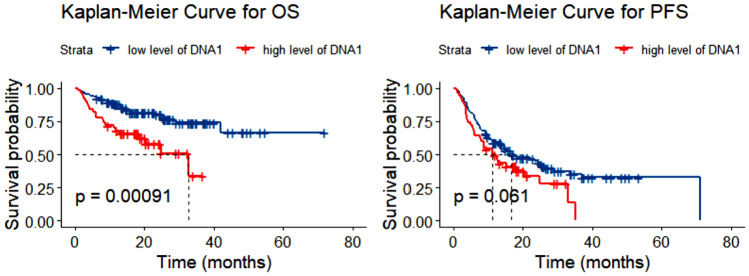
Overall survival (OS) (**A**) and progression-free survival (PFS) (**B**) in patients with PTCL according to DNA1 level. *PTCL* peripheral T-cell lymphoma, *DNA1* the EBV DNA level of patient at baseline

The entire cohort of 262 patients was included in the survival analysis. With a median follow-up of 26.6 months, the 2-year and 5-year OS rates for all cohorts were 71.2% ± 3.0% and 55.6% ± 5.0%, respectively. Regarding OS, the EBER-positive cohort showed a slightly significant difference compared to the EBER-negative cohort (*P* = 0.047; see Fig. 2A). For PFS, the EBER-positive cohort exhibited significantly poorer outcomes than the rest of the population (*P* = 0.009; see Fig. 2B). Survival outcomes were also analyzed based on the level of EBV DNA. Patients with DNA1 > 6750 copies/mL demonstrated substantially inferior OS (*P* = 0.001; see Fig. 3A) but similar PFS (*P* = 0.061; see Fig. 3B) compared to those with lower DNA1 levels. Excluding ALK + ALCL patients from the analysis, there was a statistical difference in PFS (*P* = 0.020) but not in OS (*P* = 0.100) by EBER, in OS (*P* = 0.003) but not in PFS (*P* = 0.146) by DNA1, and in both OS (*P* = 0.007) and PFS (*P* = 0.038) by DNA4.

**Fig. 4:**
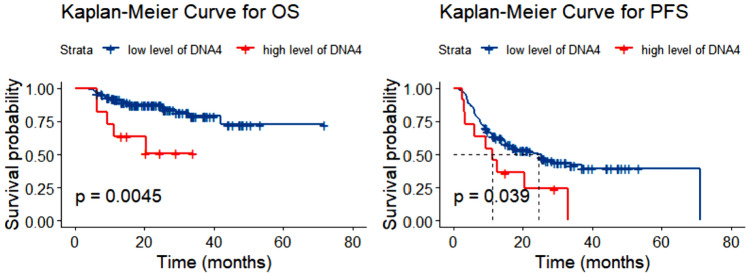
Overall survival (OS) (**A**) and progression-free survival (PFS) (**B**) in patients with PTCL according to DNA4 level. *PTCL* peripheral T-cell lymphoma, *DNA4* the EBV DNA level of patient after 4 cycles of chemotherapy

There was no difference observed between first-line treatments based on DNA4 level analysis (*P* = 0.629). Patients with higher DNA4 levels exhibited inferior OS (*P* = 0.005; see Fig. 4A) and PFS (*P* = 0.039; see Fig. 4B) compared to those with lower DNA4 levels. Furthermore, survival analysis integrating EBV status and DNA1 was conducted. Due to the small number of EBER + /DNA1- patients (*N* = 6), this group was excluded. EBER + /DNA1 + patients had the worst OS (*P* = 0.003), followed by EBER-/DNA1 + and EBER-/DNA1- patients. The final outcomes of univariate and multivariate statistical tests are presented in Table 2. In the univariate analysis, DNA1 and DNA4 were significantly associated with inferior OS. Multivariate analysis revealed that DNA4 load had an independent effect on the prognosis of PTCL for OS [hazard ratio (HR) = 5.1617; 95% confidence interval (CI) 1.1017–24.1831; *P* = 0.037]. Survival analysis of different types of PTCL by EBER is presented in Fig. S1. As AITL and PTCL-TFH were overrepresented in the EBER + group, multivariate analysis in this cohort was performed in the supplementary material. DNA1 was found to be an independent prognostic factor for OS (HR = 6.1002; 95% CI 1.0078–36.9259; *P* = 0.049) in this cohort.

**Table 2:** Univariate and multivariate statistical analysis of predictive factors for PFS and OS in PTCL Patients

Predictive factors	OS			PFS		
	Univariate analysis	Multivariate analysis		Univariate analysis	Multivariate analysis	
		HR (95% CI)	*P* value		HR (95% CI)	*P* value
Age > 60 years	0.069			0.017		
Stage III/IV	0.245			0.372		
B symptom	0.028			0.821		
BMI	0.001			0.014		
LDH > 250 U/L	0.012			0.002		
ECOG PS ≥ 2	0.000			0.000		
ENSs ≥ 2	0.115	13.3798 (2.1295–84.0657)	0.006	0.569		
WBC level	0.410			0.676		
HB level	0.000			0.006		
PLT level	0.028	0.9890 (0.9803–0.9977)	0.014	0.028		
Albumin level	0.000			0.000	0.9337 (0.8904–0.9791)	0.005
First-line treatment	0.648			0.201		
Subtype	0.283			0.083		
EBER positive	0.050			0.010		
Elevated DNA1	0.001			0.063		
Elevated DNA4	0.008	5.1617 (1.1017–24.1831)	0.037	0.043		

Some parts are blank because the data are not statistically significant in multivariate analysis

*OS* overall survival, *PFS* progression-free survival, *HR* hazard ratio, *CI* confidence interval, *BMI* bone marrow infiltration, *LDH* lactate dehydrogenase, *ECOG PS* Eastern Cooperative Oncology Group performance status, *ENSs* extranodal sites, *WBC* white blood cell, *HB* hemoglobin, PLT platelet count, *EBER* EBV-encoded RNA, *DNA1* the EBV DNA level of patient at baseline, *DNA4* the EBV DNA level of patient after 4 cycles of chemotherapy.

### A possible treatment option for EBV-associated PTCL

**Fig. 5:**
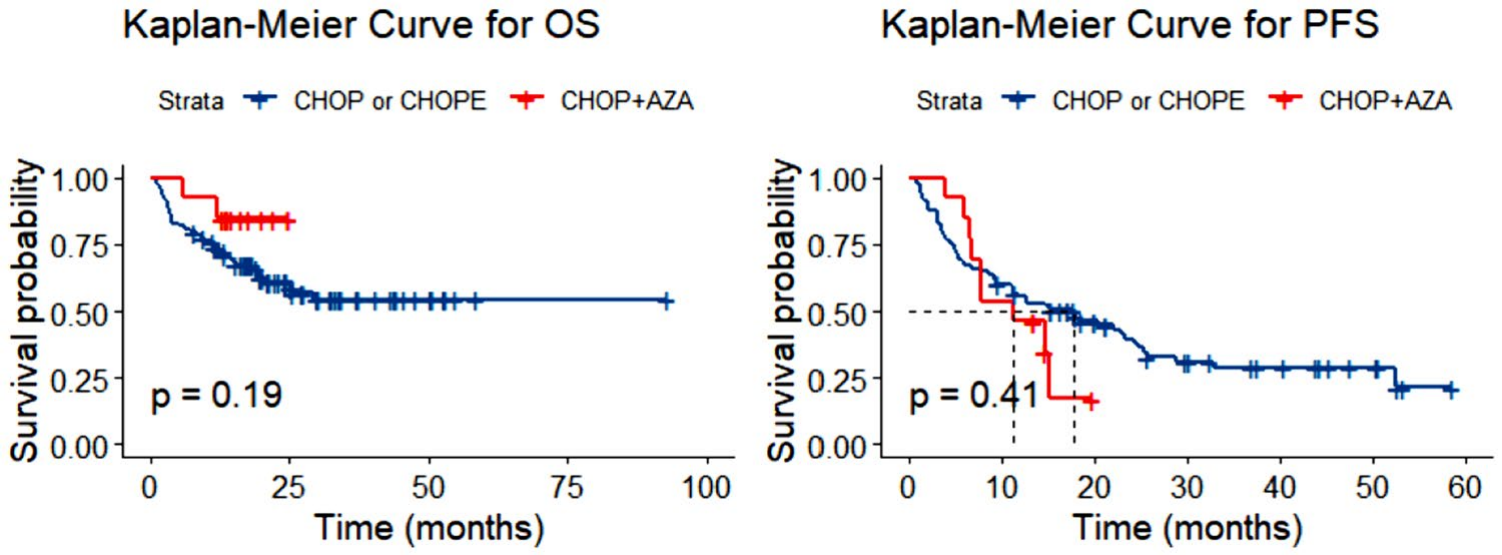
Overall survival (OS) (**A**) and progression-free survival (PFS) (**B**) in patients with EBV positive and partially positive according to different therapeutic regimen. *EBV* Epstein–Barr virus, *CHOP* cyclophosphamide, doxorubicin, vincristine, and prednisone, *CHOPE* CHOP plus etoposide, *AZA* azacytidine

For patients with EBER positivity, 3 patients received option A, 5 patients received option B, and 15 patients received option C. Although patients who received option A had the best survival outcomes (see Fig. S2), the baselines of the above three groups were not well unified (refer to Table S3). When EBER partially positive and EBER positive patients were combined, a total of 101 patients were included. After controlling for confounding factors (see Table S4), patients who received option A showed better OS (OS, *P* = 0.192, see Fig. 5A; PFS, *P* = 0.407, see Fig. 5B) compared to those with options B or C, although the differences were not statistically significant. Considering the largest population of AITL in the cohort, this population was analyzed individually. It appeared that EBV-associated AITL patients receiving option A had longer OS as well (see Fig. S3, *P* = 0.052; see Fig. S4, *P* = 0.286). Relevant confounding factors are presented in Table S5 and Table S6.

### Molecular profile

Thirty-one newly diagnosed patients were enrolled for molecular analysis. Baseline characteristics are shown in Table S7. No genetic alterations were detected in 4 patients. Common gene mutations in AITL included TET2 (73.3%), RHOA^G17V^ (46.6%), DNMT3A (33.3%), IDH2^R172^ (20.0%), and CD28 (13.3%). One case of PTCL-TFH had TET2 mutation, KRAS mutation, and PTEN mutation, while the other case of PTCL-TFH only had TP53 mutation. Mutations such as TP53 (30.0%), TET2 (40.0%), RHOA^G17V^ (30.0%), DNMT3A (20.0%), and EZH2 (10.0%) were frequent among the 10 PTCL-NOS patients. ALK genes were mutated in only 3 ALK-positive ALCL patients. The remaining ALK-negative ALCL patient developed PRDM1 and TP63 mutations.

**Fig. 6:**
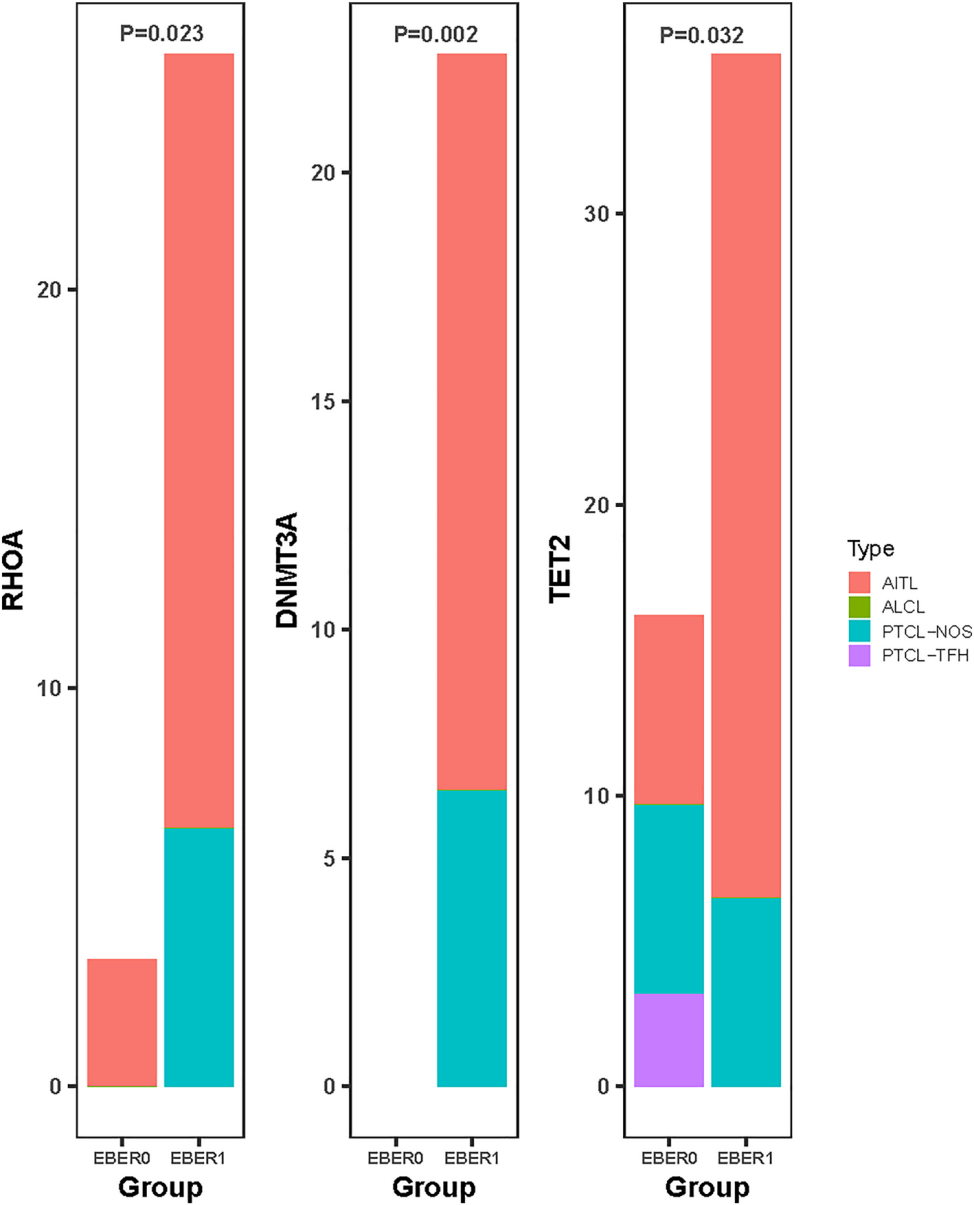
Gene mutations about RHOA^G17V^, DNMT3A or TET2 between EBER negative (EBER0) and EBER partially positive plus positive (EBER1) cohorts. The Y axis represents the proportion. *EBER* EBV-encoded RNA, *AITL* angioimmunoblastic T-cell lymphoma, *PTCL-TFH* nodal T-follicular helper (TFH) cell lymphoma, *PTCL-NOS* peripheral T-cell lymphoma, not otherwise specified, *ALCL* anaplastic large cell lymphoma

By comparing the gene levels of EBV-positive and -negative patients, no differential mutations were found. However, considering that EBER partially positive and positive patients possibly share certain characteristics, we compared them with the rest of the population (completely EBER-negative patients) and found that they were more likely to obtain DNMT3A (*P* = 0.002), RHOA^G17V^ (*P* = 0.023), and TET2 mutations (*P* = 0.032) (see Fig. 6). Baseline features and survival outcomes for patients with DNMT3A, RHOA^G17V^, or TET2 alterations are displayed in Table 3.

**Table 3:** Baseline features and survival outcomes about patients with DNMT3A, RHOA^G17V^ or TET2 mutations

Case	Subtype	EBER status	Mutations			First-line treatment	Response evaluations	OS	Times (months)
			DNMT3A	RHOA^G17V^	TET2				
1	AITL	Negative	–	–	+	Others	CR	0	24.40
2	AITL	Negative	–	+	+	Option C	PD	0	17.63
3	AITL	Partially positive	–	+	+	Option A	?	?	?
4	AITL	Partially positive	+	+	+	Option A	PD	1	11.77
5	AITL	Partially positive	–	+	+	Option A	CR	0	12.53
6	AITL	partially positive	+	+	+	Option C	CR	0	8.07
7	AITL	Partially positive	–	–	+	Option B	PD	1	19.00
8	AITL	Partially positive	+	–	+	Option C	PD	1	9.30
9	AITL	Partially positive	+	–	–	Option B	SD	0	13.40
10	AITL	Partially positive	+	+	+	Option B	?	?	?
11	AITL	Positive	–	+	+	Option A	PD	1	4.70
12	AITL	Positive	–	–	+	Others	PD	1	0.90
13	PTCL-TFH	Negative	–	–	+	Option B	PD	0	13.47
14	PTCL-NOS	Negative	–	+	–	Option C	CR	0	22.17
15	PTCL-NOS	Negative	–	–	+	Option C	SD	1	1.03
16	PTCL-NOS	Negative	–	–	+	Others	?	?	?
17	PTCL-NOS	Partially positive	+	+	+	Option C	SD	0	12.33
18	PTCL-NOS	Positive	+	+	+	Option C	PR	0	12.77

OS (1 means die and 0 means alive); ? means lost to follow-up.

*EBER*, EBV-encoded RNA, *OS*, overall survival, *AITL*, angioimmunoblastic T-cell lymphoma; *PTCL-TFH*, nodal T-follicular helper (TFH) cell lymphoma, *PTCL-NOS* peripheral T-cell lymphoma, not otherwise specified, *ALCL* anaplastic large cell lymphoma, *CR* complete remission, *PR* partial remission, *SD* stable disease, *PD* progressive disease

## Discussion

To the best of our knowledge, this is the largest study to examine the value of EBV in PTCL patients using ISH plus PCR and the first to describe the molecular characteristics of EBV-associated PTCL. We retrospectively enrolled 262 PTCL patients and compared survival outcomes based on the pretreatment EBER status, pretreatment, and post-treatment EBV DNA levels, supporting the association of EBER, DNA1, and DNA4 with survival. Our findings show that CHOP plus AZA may improve survival outcomes for EBV-associated patients. The genetic basis of PTCL revealed that EBER-positive and partially positive patients were more likely to harbor DNMT3A (*P* = 0.002), RHOA^G17V^ (*P* = 0.023), and TET2 mutations (*P* = 0.032). It is plausible that AZA reverses methylation caused by EBV infection via TET2 and/or DNMT3A mutations.

The relationship between EBER and EBV DNA has been explored extensively. Haverkos et al. 2017 found that EBER-positive patients had elevated DNA1 levels, but DNA1 failed to identify all EBER-positive patients (100% specificity; 53% sensitivity). Zhao et al. observed a high level of DNA1 associated with EBER positivity (Zhao et al. 2022). In this study, we observed poor consistency (kappa = 0.254) between EBER and DNA1, with a significant difference between EBER-positive and -negative patients in DNA1 (*P* = 0.000) but only a slight difference in DNA4 (*P* = 0.075). This indicates that EBER-positive patients tend to have higher DNA levels at diagnosis, but this tendency can change after treatment.

Our observations showed that patients with EBER negativity and lower levels of DNA1 achieved overall response rates easily (*P* = 0.015 and *P* = 0.078, respectively). However, EBER failed to predict relapse in competing risk models (*P* = 0.496), which is inconsistent with another report (Zhao et al. 2022). Overall, EBV infection impacted the disease response, but further research is still needed.

The impact of EBV on the prognosis of PTCLs has remained controversial. Previous studies have found EBV status to be either associated or not associated with a poorer prognosis (Dupuis et al. 2006; Weisenburger et al. 2011; Kim et al. 2021; Haverkos et al. 2017; Shen et al. 2022). In our study, EBER-positive patients exhibited dismal OS (*P* = 0.047) and PFS (*P* = 0.009). Univariate analysis revealed that DNA1 and DNA4 were significantly associated with inferior OS (P = 0.001; *P* = 0.005). Multivariate analysis further demonstrated that DNA4 had an independent effect on the prognosis of PTCL for OS. Consistent with previous studies by Song et al. (2022), Shen et al. (2022), and Qiu et al. (2022), EBV DNA emerged as an effective prognostic marker. It appears that EBV DNA may be superior to EBER, possibly because EBER is a qualitative diagnostic index derived from tissue samples, whereas EBV DNA is a quantitative measurement analyzed from plasma samples. This superiority of EBV DNA over EBER is reflected in the diagnostic test results: an inconsistent relationship (kappa = 0.254). Thus, monitoring EBV DNA levels may be recommended for predicting survival.

The optimal treatment regimens for PTCLs remain undefined, particularly for those with EBV infection who typically have a poor prognosis. This may be attributed to EBV infection leading to extensive methylation, resulting in immune escape and disease progression (Zhang et al. 2022; Ambinder et al. 1999). Our study found that the CHOP plus AZA regimen achieved promising OS for EBER-positive patients (Fig. S2; P = 0.046) and EBER-positive plus partially positive patients (Fig. 5; *P* = 0.192). This trend was also observed in the AITL cohort, which constituted the largest population (Fig. S3, *P* = 0.052; Fig. S4, P = 0.286). Despite the limited sample size, these cases provide insights into treatment, suggesting that EBV-infected lymphomas may respond well to hypomethylating agents. Falchi et al. conducted a phase II clinical trial combining AZA and romidepsin, where 48% of patients achieved complete remission (Falchi et al. 2021). Additionally, a patient with AITL and chronic myelomonocytic leukemia preceded by an EBER-positive large B-cell lymphoma showed a favorable response to AZA (Saillard et al. 2017). These findings resonate with our results, supporting the notion that targeting DNA methylation abnormalities could be an effective strategy for patients with EBV-associated lymphomas.

The molecular paradigm of EBV was also investigated. We observed that EBER-positive and partially positive patients were more likely to harbor DNMT3A (*P* = 0.002), RHOA^G17V^ (*P* = 0.023), and TET2 mutations (*P* = 0.032) (Fig. 6). Previous reports have indicated a correlation between TET2 and/or DNMT3A mutations and DNA methylation in hematological tumors (Xie et al. 2020; Lemonnier et al. 2018, Woods and Levine 2015). However, the association of EBV with methylation in PTCL has not been extensively studied. Considering the observed clinical benefit of AZA in EBV-related patients, it is conceivable that hypomethylating agents like AZA could target methylation caused by EBV through DNMT3A or TET2 to improve prognosis. Nonetheless, the detailed genetic features of EBV in PTCL remain unclear. Further studies with larger cohorts are warranted to explore this aspect, especially considering the partial overlap between the subjects of survival analysis and genetic analysis in our study.

Several limitations should be acknowledged in our study. Firstly, its retrospective nature and single-center design with varied therapeutic regimens may introduce bias, despite efforts to mitigate this through comparable clinical characteristics and statistical adjustments. Secondly, the limited sample size of EBV-infected or AZA-treated patients warrants validation in larger prospective cohorts with homogenous treatment approaches. Thirdly, while molecular analysis related to DNA methylation was conducted, there was not complete consistency between the subjects of survival analysis and genetic analysis.

In conclusion, EBER positivity, elevated DNA1, and elevated DNA4 were significantly associated with inferior survival, highlighting the importance of monitoring EBV DNA in newly diagnosed PTCL. Front-line treatment with CHOP plus AZA is possibly good for EBER partially positive and positive patients, who may exhibit high levels of methylation due to DNMT3A or TET2 mutations. However, these speculations require further exploration in larger cohort studies.

## Supplementary Information

Below is the link to the electronic supplementary material.

Supplementary file1 (DOCX 7865 KB)

## Data Availability

Not applicable.
