# Supplementary material for: Role of plasma EBV-DNA load and EBER status on newly diagnosed peripheral T-cell lymphoma
Source: J Cancer Res Clin Oncol. 2024 Apr 8;150(4):181. doi: 10.1007/s00432-024-05702-9 (PMC11001675; doi:10.1007/s00432-024-05702-9)
Supplement: Supplementary file 1 — Supplementary file1 (DOCX 7865 KB) [file 432_2024_5702_MOESM1_ESM.docx]

**Content**

[The inclusion criteria for survival analysis 1](#_Toc22463)

[The inclusion criteria for molecular analysis 2](#_Toc15193)

[The exclusion criteria for survival analysis 2](#_Toc25946)

[Samples size 2](#_Toc21145)

[Description of data 2](#_Toc19456)

[The panel of 103 genes used in NGS 3](#_Toc21391)

[Survival analysis of different subtypes 3](#_Toc31751)

[Table. Univariate and multivariate statistical analysis of predictive factors for PFS and OS in AITL plus PTCL-TFH Patients* 4](#_Toc13794)

[Supplementary table 5](#_Toc23674)

[Table S1. Diagnostic performance in all PTCL patients 5](#_Toc25193)

[Table S2. Response evaluations by EBV status 5](#_Toc16610)

[Table S3. Baseline characteristics for EBER-positive PTCL patients by three treatment options 6](#_Toc31829)

[Table S4. Baseline characteristics for PTCL patients including EBER partially positive and EBER positive cases by treatment options 6](#_Toc3667)

[Table S5. Baseline characteristics for EBV-positive AITL patients by treatment options 7](#_Toc28803)

[Table S6. Baseline characteristics for AITL patients including EBER partially positive and EBER positive cases by treatment options 7](#_Toc4584)

[Table S7. Baseline characteristics for PTCL patients who underwent NGS 8](#_Toc25742)

[Supplementary figure 9](#_Toc23567)

[Figure S1. Overall survival (OS) and progression-free survival (PFS) by EBV status according to different subtypes 9](#_Toc11104)

[Figure S2. Overall survival (OS, P =0.046) and progression-free survival (PFS, P =0.104) in patients with EBV positive according to different therapeutic regimen 10](#_Toc20316)

[Figure S3. Overall survival (OS, P =0.052) and progression-free survival (PFS, P =0.281) in AITL patients with EBV positive according to different therapeutic regimen 11](#_Toc21326)

[Figure S4. Overall survival (OS, P =0.286) and progression-free survival (PFS, P =0.529) in AITL patients with EBV positive and partially positive according to different therapeutic regimen 11](#_Toc18100)

**The inclusion criteria for survival analysis**

1. Patients with newly diagnosed and histologically confirmed PTCL including PTCL-NOS, AITL, PTCL-TFH and sALCL;
2. Patients who underwent more than 6 months of follow-up;
3. Patients who had pre-treatment EBER measurement.

**The inclusion criteria for molecular analysis**

1. Patients with newly diagnosed and histologically confirmed PTCL including PTCL-NOS, AITL, PTCL-TFH and sALCL;
2. Patients who underwent pre-treatment next generation sequencing.
3. Patients who had pre-treatment EBER measurement.

**The exclusion criteria for survival analysis**

1. Patients who had other uncured malignancy cancers or severe infection before enrollment.
2. The primary cutaneous ALCL, and breast-implant-associated ALCL were excluded.
3. Patients who gave up treatment because of financial condition

**Samples size**

The whole 262 patients were included in survival analysis and 31 patients were enrolled for molecular analysis.

**Description of data**

Age (>60 year), stage(I/II or III/IV), symptom (A or B), BMI (yes or no), LDH (>250U/L), ECOG PS (≥2), ENSs (≥2), EBER (positive or negative), DNA1 (high level or low level) and DNA4 (high level or low level) are categorical variables.

Continuous variables were transformed into categorical variables by ROC analysis ( DNA1 and DNA4) or reports from relative literature (Age>60 year and LDH>250U/L are used for IPI score; EBER is a qualitative indicator using a cut-off value of 50% from relative literature).

WBC, HB, PLT and Albumin are continuous variables.

**The panel of 103 genes used in NGS**

| ALK | APC | ARID1A | ARID1B | ARID2 | ATM | ATR | B2M |
| --- | --- | --- | --- | --- | --- | --- | --- |
| BCL11B | BCOR | BCORL1 | BRAF | CARD11 | CBLB | CD28 | CD58 |
| CDC73 | CDKN1B | CDKN1C | CDKN2A | CDKN2B | CDKN2C | CHD8 | CIITA |
| CREBBP | CTCF | CTNNB1 | CUX1 | DDX3X | DNM2 | DNMT3A | DUSP22 |
| ECSIT | EP300 | ETV6 | EZH2 | FAS | FAT1 | FBXW7 | FLT3 |
| FOXO1 | GATA1 | GATA2 | GATA3 | GNA13 | GNAS | IDH1 | IDH2 |
| IL7R | IRF4 | JAK1 | JAK2 | JAK3 | KDM6A | KIT | KMT2A |
| KMT2B | KMT2C | KMT2D | KRAS | MAP2K1 | MED12 | MTOR | MYC |
| NF1 | NOTCH1 | NOTCH2 | NPM1 | NRAS | PDGFRA | PDGFRB | PHF6 |
| PIK3CA | POT1 | PRDM1 | PTEN | PTPN2 | RAD21 | RB1 | RHOA |
| RUNX1 | SETD2 | SMARCA4 | SMO | SPEN | STAT3 | STAT5A | STAT5B |
| STAT6 | STIL | SUZ12 | SYK | TBL1XR1 | TCL1A | TET2 | TNFAIP3 |
| TNFRSF14 | TP53 | TP63 | TP73 | TRAF3 | WT1 | ZAP70 |  |

**Survival analysis of different subtypes**

The survival outcomes of 147 AITL patients, 19 PTCL-TFH patients, 56 PTCL-NOS patients and 40 sALCL patients were analyzed separately. Patients with AITL (14.2%) and PTCL-TFH (15.7%) were more likely to have EBV infection compare with those with PTCL-NOS (8.9%) and sALCL (2.5%). The P values reflecting OS between EBER-negative and EBER-positive among AITL, PTCL-TFH, PTCL-NOS, and sALCL were 0.069, 0.385, 0.671 and 0.000, respectively. For PFS, the P values among AITL, PTCL-TFH, PTCL-NOS, and sALCL were 0.032, 0.509, 0.707 and 0.000, respectively (**Figure S1**).

AITL (14.3%) and PTCL-TFH (15.8%) are highly associated with EBV infection. In this scenario, multivariate analysis of survival was performed in the cohort. Moreover, EBV-associated AITL had longer OS after receiving CHOP plus AZA regimen than the counterparts (**Figure S3** and **S4**). Relative confounding factors had been well controlled

The reasons for this difference may be: (1) PTCL is highly heterogeneous, and the genetic profiles of each subtype are different, which affects the prognosis of the disease; (2) In addition to EBV, there are other factors, such as the expression of CD30, that affect the prognosis of PTCL; (3) After dividing into subgroups, the small sample size in each group may lead to false negative results.

**Table.** Univariate and multivariate statistical analysis of predictive factors for PFS and OS in AITL plus PTCL-TFH Patients*

| Predictive factors | OS | | | PFS | | |
| --- | --- | --- | --- | --- | --- | --- |
|  | Univariate analysis | Multivariate analysis | | Univariate analysis | Multivariate analysis | |
|  |  | HR (95%CI) | P-value |  | HR (95%CI) | P-value |
| Age >60 years | 0.235 |  |  | 0.046 |  |  |
| Stage III/IV | 0.207 |  |  | 0.670 |  |  |
| B symptom | 0.008 |  |  | 0.742 |  |  |
| BMI | 0.000 |  |  | 0.001 |  |  |
| LDH >250U/L | 0.390 |  |  | 0.157 |  |  |
| ECOG PS ≥2 | 0.000 |  |  | 0.006 |  |  |
| ENSs ≥2 | 0.078 |  |  | 0.051 | 7.9075  (1.5492-40.362) | **0.013** |
| WBC level | 0.860 |  |  | 0.555 |  |  |
| HB level | 0.000 | 1.0530  (1.0146-0.1.0929) | **0.006** | 0.0030 |  |  |
| PLT level | 0.065 | 0.9669  (0.9500-0.9842) | **0.000** | 0.007 | 0.9944  (0.9908-0.9980) | **0.002** |
| Albumin level | 0.000 |  |  | 0.004 |  |  |
| First-line treatment | 0.964 |  |  | 0.425 |  |  |
| EBER positive | 0.132 |  |  | 0.036 |  |  |
| Elevated DNA1 | 0.000 | 6.1002  (1.0078-36.9259) | **0.049** | 0.042 |  |  |
| Elevated DNA4 | 0.073 |  |  | 0.114 |  |  |

Notes: *Some parts are blank because the data is not statistically significant in multivariate analysis.

Abbreviations: AITL, angioimmunoblastic T-cell lymphoma; PTCL-TFH, nodal T-follicular helper (TFH) cell lymphoma; OS, overall survival; PFS, progression-free survival; HR, hazard ratio; CI, confidence interval; BMI, bone marrow infiltration; LDH, lactate dehydrogenase; ECOG PS, Eastern Cooperative Oncology Group performance status; ENSs, extranodal sites; WBC, white blood cell; HB, hemoglobin; PLT, platelet count; EBER, EBV-encoded RNA; DNA1, the EBV DNA level of patient at baseline; DNA4, the EBV DNA level of patient after 4 cycles of chemotherapy.

**Supplementary table**

**Table S1.** Diagnostic performance in all PTCL patients

| PTCL | | EBER | | kappa |
| --- | --- | --- | --- | --- |
|  |  | (+) | (-) |  |
| EBV DNA | (+) | 13 | 37 | 0.254 |
|  | (-) | 6 | 111 |  |
| Sensitivity=68.4% Specificity=75.0% | | | | |

Abbreviations: PTCL, peripheral T-cell lymphoma; EBER, EBV-encoded RNA; EBV, Epstein-Barr virus.

**Table S2.** Response evaluations by EBV status

| Response evaluations | ORR | SD | PD | P-value for ORR |
| --- | --- | --- | --- | --- |
| EBER-negative | 51.3% | 14.2% | 34.5% | 0.015 |
| EBER-positive | 30.0% | 33.3% | 36.7% |  |
| High level of DNA1 | 38.0% | 26.0% | 36.0% | 0.078 |
| Low level of DNA1 | 51.7% | 12.7% | 35.6% |  |

Abbreviations: EBV, Epstein-Barr virus; ORR, overall response rate; SD, stable disease; PD, progressive disease. EBER, EBV-encoded RNA; DNA1, the EBV DNA level of patient at baseline.

**Table S3.** Baseline characteristics for EBER-positive PTCL patients by three treatment options

| **Characteristic** | **Option A**  **(n=3)** | **Option B**  **(n=5)** | **Option C**  **(n=15)** | **P-value** |
| --- | --- | --- | --- | --- |
| **Age >60 years, no. (%)** | 2 (66.7) | 2 (40) | 2 (13.3) | 0.115 |
| **Stage III/IV, no. (%)** | 3 (100) | 3 (60) | 15 (100) | **0.019** |
| **ENSs ≥2, no. (%)** | 0 (0) | 0 (0) | 0 (0) | 1.000 |
| **B symptom, no. (%)** | 2 (66.7) | 4 (80.0) | 7 (46.7) | 0.399 |
| **BMI, no. (%)** | 1 (33.3) | 0 (0) | 4 (26.7) | 0.399 |
| **ECOG PS ≥2, no. (%)** | 0 (0) | 2 (40) | 8 (53.3) | 0.232 |
| **Elevated LDH, no. (%)** | 1 (33.3) | 4 (80.0) | 9 (64.3) | 0.412 |
| **HB (median[range], g/L)** | 128.0 (87.0 -131.0) | 110.0 (52.0-121.0) | 113.0 (59.0-136.0) | 0.636 |
| **PLT (median[range], 109 /L)** | 161.0 (147.0-213.0) | 150.5 (30.0-225.0) | 147.0 (18.0-408.0) | 0.713 |
| **Albumin (median[range], g/L)** | 43.2 (42.8-46.0) | 34.7 (1.2-43.3) | 35.2 (23.9-45.9) | 0.058 |
| **Elevated DNA1, no. (%)** | 0 (0) | 2 (50) | 7 (46.7) | **0.008** |
| **Elevated DNA4, no. (%)** | 0 (0) | 1 (25) | 0 (0) | 0.495 |

Abbreviations: PTCL, peripheral T-cell lymphoma; EBER, EBV-encoded RNA; ENSs, extranodal sites; BMI, bone marrow infiltration; ECOG PS, Eastern Cooperative Oncology Group performance status; LDH, lactate dehydrogenase; HB, hemoglobin; PLT, platelet count; SD, standard deviation; DNA1, the EBV DNA level of patient at baseline; DNA4, the EBV DNA level of patient after 4 cycles of chemotherapy.

**Table S4.** Baseline characteristics for PTCL patients including EBER partially positive and EBER positive cases by treatment options

| **Characteristic** | **Option A**  **(n=13)** | **Option B+C**  **(n=88)** | **P-value** |
| --- | --- | --- | --- |
| **Age >60 years, no. (%)** | 9 (69.2) | 59 (67.0) | 1.000 |
| **Stage III/IV, no. (%)** | 13 (100) | 80 (91.0) | 0.592 |
| **ENSs ≥2, no. (%)** | 1 (7.7) | 1 (1.1% | 0.242 |
| **B symptom, no. (%)** | 5 (38.5) | 49 (55.7) | 0.372 |
| **BMI, no. (%)** | 4 (30.8) | 19 (21.6) | 0.486 |
| **ECOG PS ≥2, no. (%)** | 2 (15.4) | 24 (27.3) | 0.506 |
| **Elevated LDH, no. (%)** | 9 (69.2) | 51 (60.0) | 0.761 |
| **HB (median ± SD, g/L)** | 101.7 ± 19.3 | 103.7 ± 27.1 | 0.800 |
| **PLT (median ± SD, 109 /L)** | 167.9 ± 78.8 | 181.8 ± 108.7 | 0.660 |
| **Albumin (median ± SD, g/L)** | 40.2 ± 4.7 | 36.4 ± 7.9 | 0.098 |
| **Elevated DNA1, no. (%)** | 6 (46.2) | 28 (50.0) | 1.000 |
| **Elevated DNA4, no. (%)** | 0 (0) | 7 (18.4) | 0.318 |

Abbreviations: PTCL, peripheral T-cell lymphoma; EBER, EBV-encoded RNA; ENSs, extranodal sites; BMI, bone marrow infiltration; ECOG PS, Eastern Cooperative Oncology Group performance status; LDH, lactate dehydrogenase; HB, hemoglobin; PLT, platelet count; SD, standard deviation; DNA1, the EBV DNA level of patient at baseline; DNA4, the EBV DNA level of patient after 4 cycles of chemotherapy.

**Table S5.** Baseline characteristics for EBV-positive AITL patients by treatment options

| **Characteristic** | **Option A**  **(n=3)** | **Option B+C**  **(n=12)** | **P-value** |
| --- | --- | --- | --- |
| **Age >60 years, no. (%)** | 1 | 10 | 0.154 |
| **Stage III/IV, no. (%)** | 3 | 12 | 1.000 |
| **ENSs ≥2, no. (%)** | 0 | 0 | 1.000 |
| **B symptom, no. (%)** | 2 | 6 | 0.554 |
| **BMI, no. (%)** | 1 | 3 | 1.000 |
| **ECOG PS ≥2, no. (%)** | 0 | 6 | 0.229 |
| **Elevated LDH, no. (%)** | 1 | 6 | 1.000 |
| **HB (median[range], g/L)** | 115.3 (87.0-131.0) | 97.3 (59.0-136.0) | 0.564 |
| **PLT (median[range], 10^9^ /L)** | 173.7 (147.0-213.0) | 162.4 (18.0-408.0) | 0.312 |
| **Albumin (median[range], g/L)** | 44.0 (42.8-46.0) | 34.2 (23.9-45.9) | **0.021** |
| **Elevated DNA1, no. (%)** | 0 | 6 | **0.012** |
| **Elevated DNA4, no. (%)** | 0 | 1 | 1.000 |

Abbreviations: AITL, angioi mmunoblastic T-cell lymphoma; EBER, EBV-encoded RNA; ENSs, extranodal sites; BMI, bone marrow infiltration; ECOG PS, Eastern Cooperative Oncology Group performance status; LDH, lactate dehydrogenase; HB, hemoglobin; PLT, platelet count; SD, standard deviation; DNA1, the EBV DNA level of patient at baseline; DNA4, the EBV DNA level of patient after 4 cycles of chemotherapy.

**Table S6.** Baseline characteristics for AITL patients including EBER partially positive and EBER positive cases by treatment options

| **Characteristic** | **Option A**  **(n=12)** | **Option B+C**  **(n=66)** | **P-value** |
| --- | --- | --- | --- |
| **Age >60 years, no. (%)** | 20 | 46 | 1.000 |
| **Stage III/IV, no. (%)** | 12 | 61 | 1.000 |
| **ENSs ≥2, no. (%)** | 1 | 1 | 0.286 |
| **B symptom, no. (%)** | 5 | 36 | 0.534 |
| **BMI, no. (%)** | 4 | 15 | 0.472 |
| **ECOG PS ≥2, no. (%)** | 2 | 17 | 0.720 |
| **Elevated LDH, no. (%)** | 8 | 35 | 0.540 |
| **HB (median ± SD, g/L)** | 101.9 ± 20.1 | 102.8 ± 27.3 | 0.873 |
| **PLT (median ± SD, 10^9^ /L)** | 165.8 ± 81.8 | 177.0 ± 98.9 | 0.766 |
| **Albumin (median ± SD, g/L)** | 40.1 ± 4.9 | 37.2 ± 7.1 | 0.062 |
| **Elevated DNA1, no. (%)** | 6 | 21 | 1.000 |
| **Elevated DNA4, no. (%)** | 0 | 6 | 0.304 |

Abbreviations: AITL, angioimmunoblastic T-cell lymphoma; EBER, EBV-encoded RNA; ENSs, extranodal sites; BMI, bone marrow infiltration; ECOG PS, Eastern Cooperative Oncology Group performance status; LDH, lactate dehydrogenase; HB, hemoglobin; PLT, platelet count; SD, standard deviation; DNA1, the EBV DNA level of patient at baseline; DNA4, the EBV DNA level of patient after 4 cycles of chemotherapy.

**Table S7.** Baseline characteristics for PTCL patients who underwent NGS

| **Characteristic** | **Total**  **(n=31)** |
| --- | --- |
| **Age >60 years, no. (%)** | 15 (48.4) |
| **Stage III/IV, no. (%)** | 29 (93.5) |
| **ENSs ≥2, no. (%)** | 5 (16.1) |
| **B symptom, no. (%)** | 15 (48.4) |
| **BMI, no. (%)** | 6 (19.4) |
| **ECOG PS ≥2, no. (%)** | 10 (32.3) |
| **Elevated LDH, no. (%)** | 18 (60.0) |
| **EBER positive, no. (%)** | 4 (12.9) |
| **EBER partially positive, no. (%)** | 11 (35.4) |
| **Subtype, no. (%)** |  |
| AITL | 15 (48.4) |
| PTCL-TFH | 2 (6.5) |
| PTCL-NOS | 10 (32.3) |
| ALCL | 4 (12.9) |
| **Gene mutations, no. (%)** |  |
| DNMT3A | 7 (22.6) |
| IDH2 | 4 (12.9) |
| RHOA | 10 (32.3) |
| TET2 | 16 (51.6) |
| TP53 | 5 (16.1) |
| ALK | 3 (9.7) |
| STAT3 | 2 (6.5) |
| KMT2D | 3 (9.7) |
| CD28 | 3 (9.7) |

Notes: Partial items like LDH and have missing values

Abbreviations: PTCL, peripheral T-cell lymphoma; ENSs, extranodal sites; BMI, bone marrow infiltration; ECOG PS, Eastern Cooperative Oncology Group performance status; LDH, lactate dehydrogenase; EBER, EBV-encoded RNA; AITL, angioimmunoblastic T-cell lymphoma; PTCL-TFH, nodal T-follicular helper (TFH) cell lymphoma; PTCL-NOS, peripheral T-cell lymphoma, not otherwise specified; ALCL, anaplastic large cell lymphoma.

**Supplementary figure**

**Fig. S1** Overall survival (OS) and progression-free survival (PFS) by EBV status according to different subtypes

| 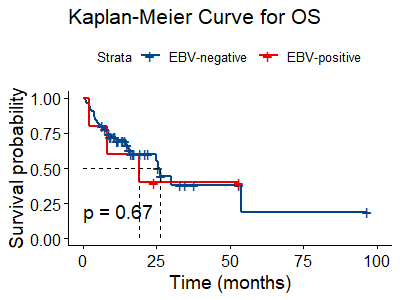 | 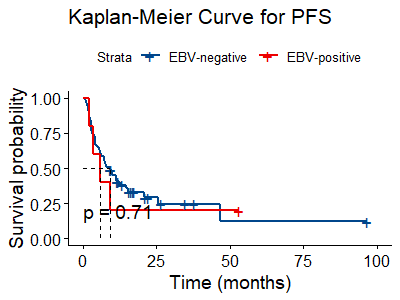 |
| --- | --- |
| ( PTCL-NOS) | ( PTCL-NOS) |
| 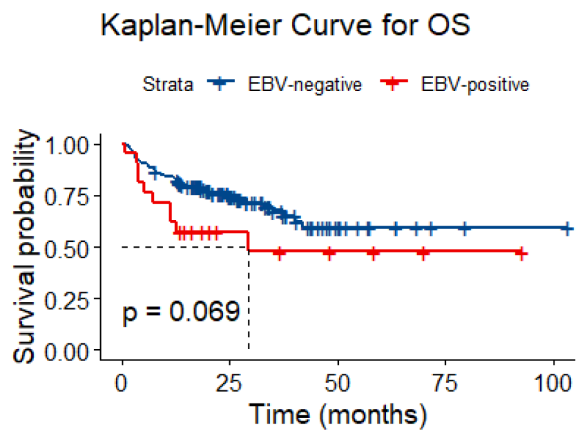 | 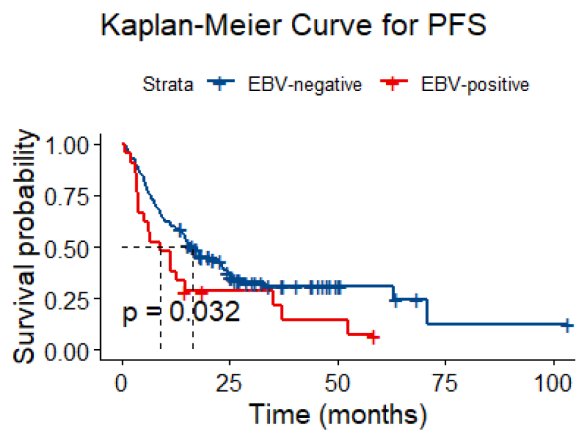 |
| (AITL) | (AITL) |
| 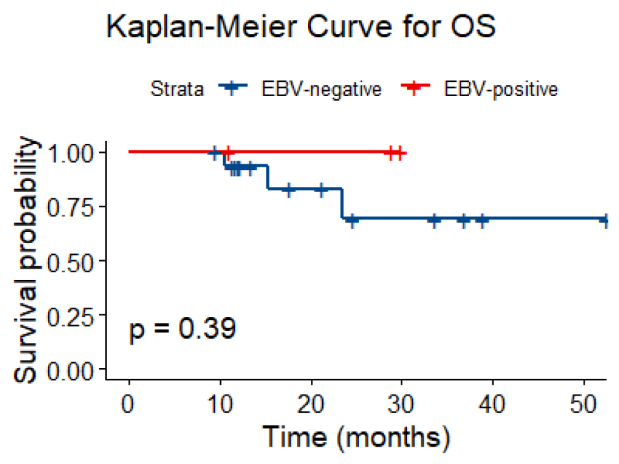 | 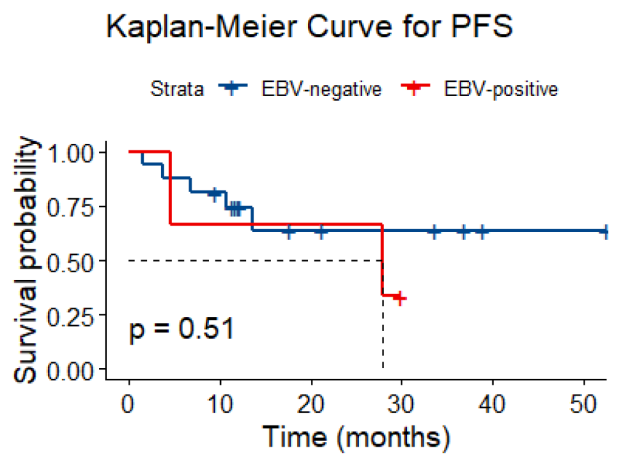 |
| (PTCL-TFH) | (PTCL-TFH) |
| 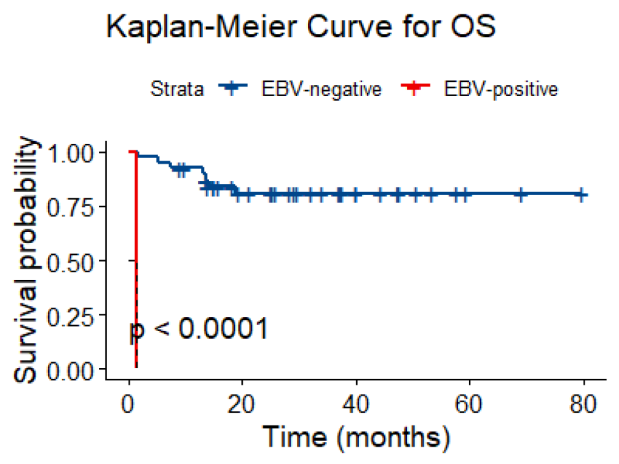 | 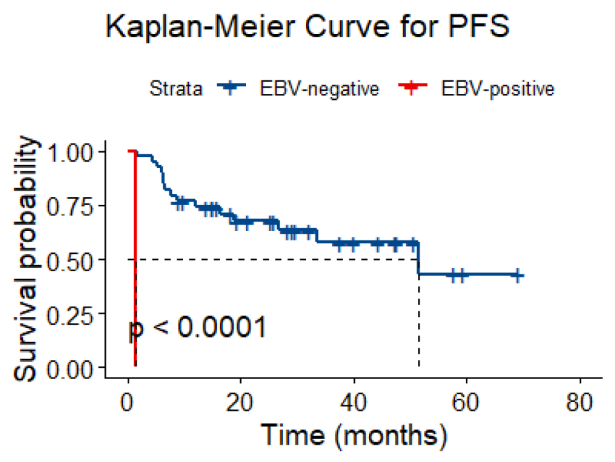 |
| (ALCL) | (ALCL) |

Abbreviations:EBV, Epstein-Barr virus; PTCL-NOS, peripheral T-cell lymphoma, not otherwise specified; AITL, angioimmunoblastic T-cell lymphoma; PTCL-TFH, nodal T-follicular helper (TFH) cell lymphoma; ALCL, anaplastic large cell lymphoma;

**Fig. S2** Overall survival (OS, P =0.046) and progression-free survival (PFS, P =0.104) in patients with EBV positive according to different therapeutic regimen


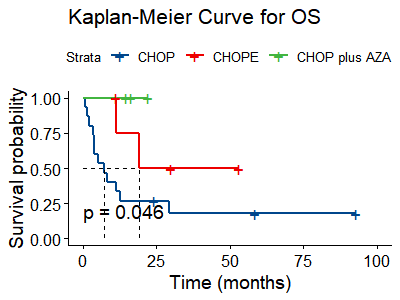

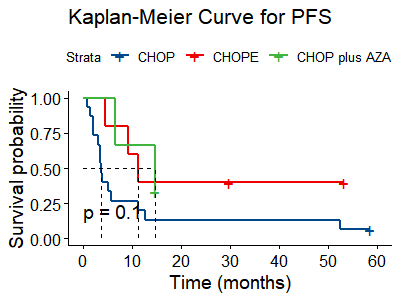


Abbreviations: EBV: Epstein-Barr virus; CHOP, cyclophosphamide, doxorubicin, vincristine, and prednisone; CHOPE, CHOP plus etoposide; AZA, azacytidine.

**Fig. S3** Overall survival (OS, P =0.052) and progression-free survival (PFS, P =0.281) in AITL patients with EBV positive according to different therapeutic regimen


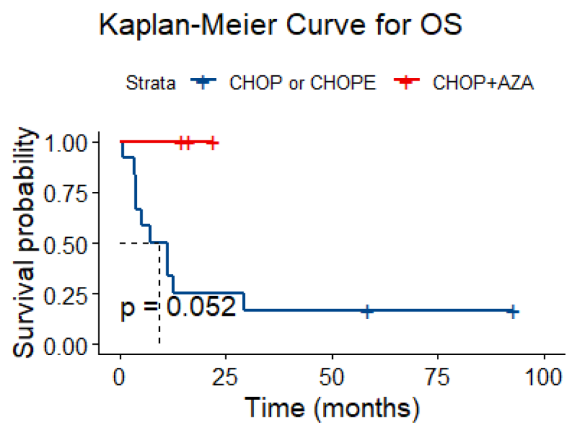

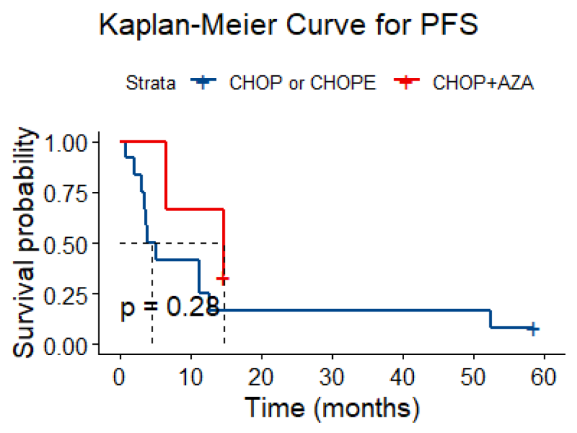


Abbreviations: AITL, angioimmunoblastic T-cell lymphoma; EBV: Epstein-Barr virus; CHOP, cyclophosphamide, doxorubicin, vincristine, and prednisone; CHOPE, CHOP plus etoposide; AZA, azacytidine.

**Fig. S4** Overall survival (OS, P =0.286) and progression-free survival (PFS, P =0.529) in AITL patients with EBV positive and partially positive according to different therapeutic regimen


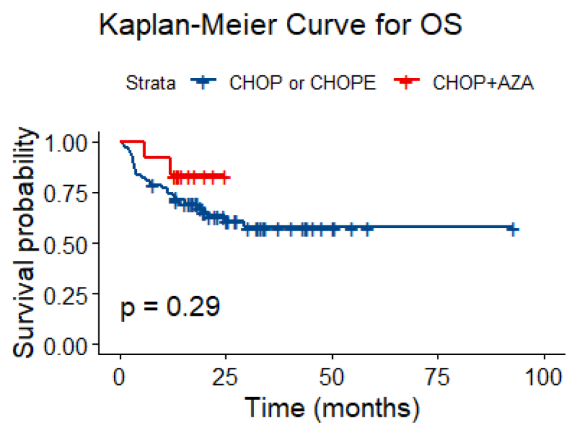

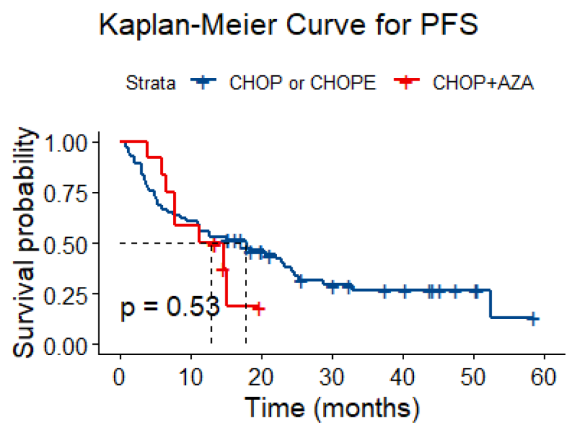


Abbreviations: AITL, angioimmunoblastic T-cell lymphoma; EBV: Epstein-Barr virus; CHOP, cyclophosphamide, doxorubicin, vincristine, and prednisone; CHOPE, CHOP plus etoposide; AZA, azacytidine.
